# Evaluation of effectiveness of protein expression of DNA vaccine in CHO Cells

**DOI:** 10.1186/1753-6561-8-S4-P142

**Published:** 2014-10-01

**Authors:** Alexandre Brum, Andrea Rezende, Alex Rodrigues, Henrique Angelo, Carlos Reis, Thais Collares, Claudia Hartleben, Vasco Azevedo, Sibele Borsuk

**Affiliations:** 1 UFPel - biotecnologia - LPDI Laboratório de pesquisa em doenças infecciosas, Pelotas, Brazil; 2 UFPel - biotecnologia - Laboratório de imunodiagnóstico, Pelotas, Brazil; 3 UFMG - departamento de biologia geral, Belo Horizonte, Brazil

## 

The caseous lymphadenitis (CLA) is a common disease that affects ruminants around the world. The CLA is caused by gram-positive bacteria known as *Corynebacterium pseudotuberculosis*, which is an intracellular facultative pathogen. In Brazil, the high prevalence occurs principally in northeast and southwest, where has the major herd of sheep and goat causing important economic losses. The actual commercial vaccine confers some level of protection and as based in attenuated or inactive bacteria. The third generation of vaccine includes the DNA vaccine, which can give some advantages as does not cause infection and the stability [1-3]. Thus the aim of this study was develop and evaluate DNA vaccines based on genes that encode for secreted proteins of *C. pseudotuberculosis*. For this, we cloned on eukaryotic expression pTARGET vector, gene fragments of *C. pseudotuberculosis *namely *Cp1002_1957 *and *Cp1002_1802*, which encoded for the LipY protein and transferase B protein, respectively. After PCR using specific primers to each gene, the encode sequence were ligated on pTARGET vector (Promega) and the ligation reaction was used to transform *Escherichia coli *Top10 by eletroporation. The transformation was plated in LB with ampicilin and X-gal substrate. After incubation, the white colonies were select for a screening by digestion with the enzyme *Eco*RI to confirm gene insertion on vector. Five clones of each gene are selected to transfection of the CHO cells in order to confirm the expression level of proteins by indirect immunofluorescence using sera of mice immunized with the recombinant proteins (rCP1957 and rCP1802). The immunofluorescence analysis show high levels of protein expression (CP1957 and Cp1802) in CHO cells in all clones tested. Both DNA vaccines (pTARGET/1802 and pTARGET/1957) are able to express the proteins in CHO cells in vitro. On the next step, we will perform the animal immunization to confirm the protection level (challenge test) and the immunomodulatory response.
